# Do trees starve to death? Insights into the roles of carbon reserves and stem photosynthesis in drought-induced sapling mortality

**DOI:** 10.1093/plphys/kiag532

**Published:** 2026-08-18

**Authors:** Laura Fernández-de-Uña

**Affiliations:** Assistant Features Editor, Plant Physiology, American Society of Plant Biologists; Department of Plant Biology and Soil Sciences, Universidade de Vigo, Ourense 32004, Spain

Increasing temperatures and drought (ie, water scarcity) place significant stress on plants. Rising temperatures elevate the atmospheric vapor pressure deficit, increasing plant water demand at a time when drought episodes are becoming more frequent globally, including moist tropical forests. Changes in climatic conditions can alter forest productivity and health and lead to an increase in tree mortality rates (Hammond et al 2022; McDowell et al 2022).

Plants cope with drought episodes by closing stomata. Stomatal closure reduces water loss and regulates water potential (Ψ; ie, water energy, including pressure) in the xylem (ie, the plant's water-conductive tissue). High pressures in xylem conduits (ie, more negative Ψ) facilitate the formation of air bubbles and, in turn, increase the risk of xylem embolism, which disrupts water transport to the canopy. Plant species differ in the promptness of stomatal closure, either closing stomata early in the drought, in turn limiting CO_2_ uptake, or later on, thus facing greater risk of xylem embolism. Regardless of the strategy followed, severe drought, in terms of intensity and/or length, may eventually cause plant death. Two main, nonmutually exclusive mechanisms have been proposed to drive tree mortality under drought. Trees may die from hydraulic failure when extensive embolism damage leads to the complete desiccation of the plant (McDowell et al 2008, 2022). Conversely, the carbon starvation hypothesis posits that the reduction in carbon uptake as a result of stomatal closure causes the plant to starve due to the consumption of stored nonstructural carbohydrates (NSCs; ie, water-soluble sugars and starch) for metabolic, hydraulic, or defense functions, including the osmotic regulation of Ψ (McDowell et al 2008, 2022; Sala et al 2012; Tomasella et al 2020). While drought-induced hydraulic failure has been abundantly observed, evidence in support of the carbon starvation hypothesis is elusive (Adams et al 2017). Greater NSC reserves have occasionally been associated with longer plant survival under drought (O’Brien et al 2014), and carbon reserves have often been found to decrease toward the time of death (Adams et al 2017), but NSC depletion has rarely been observed (Sevanto et al 2014). This lack of support for the carbon starvation hypothesis is likely due to the high interdependence between carbon and hydraulic constraints during drought and the multiple factors affecting carbon balance in plants.

Recently in *Plant Physiology*, Ávila-Lovera et al (2026) tested the role of carbon starvation on tropical tree mortality using saplings of *Calophyllum longifolium*. Some species, including *C. longifolium* saplings, are able to assimilate atmospheric CO_2_ and/or reuse the CO_2_ respired by inner tissues (eg, wood) in green stems thanks to the presence of photosynthetic tissues in the living bark. Therefore, they additionally tested whether stem photosynthesis played a role in buffering carbon reserve depletion during drought.

First, the authors exposed half of their saplings to increased CO_2_ concentrations in order to enhance NSC concentrations in saplings (Fig. 1). The experiment successfully increased starch and total NSC concentrations in leaves, stem bark, and, to a lesser extent, stem wood in trees subjected to elevated CO_2_ compared with control ones. Other traits were unaffected by the treatment, including predawn water potential (Ψ_PD_, indicative of the plant water status), leaf and stem photosynthetic rates (a measure of CO_2_ uptake), stomatal conductance (g_s_, a measure of stomatal aperture), and plant height and biomass.

**Figure 1:**
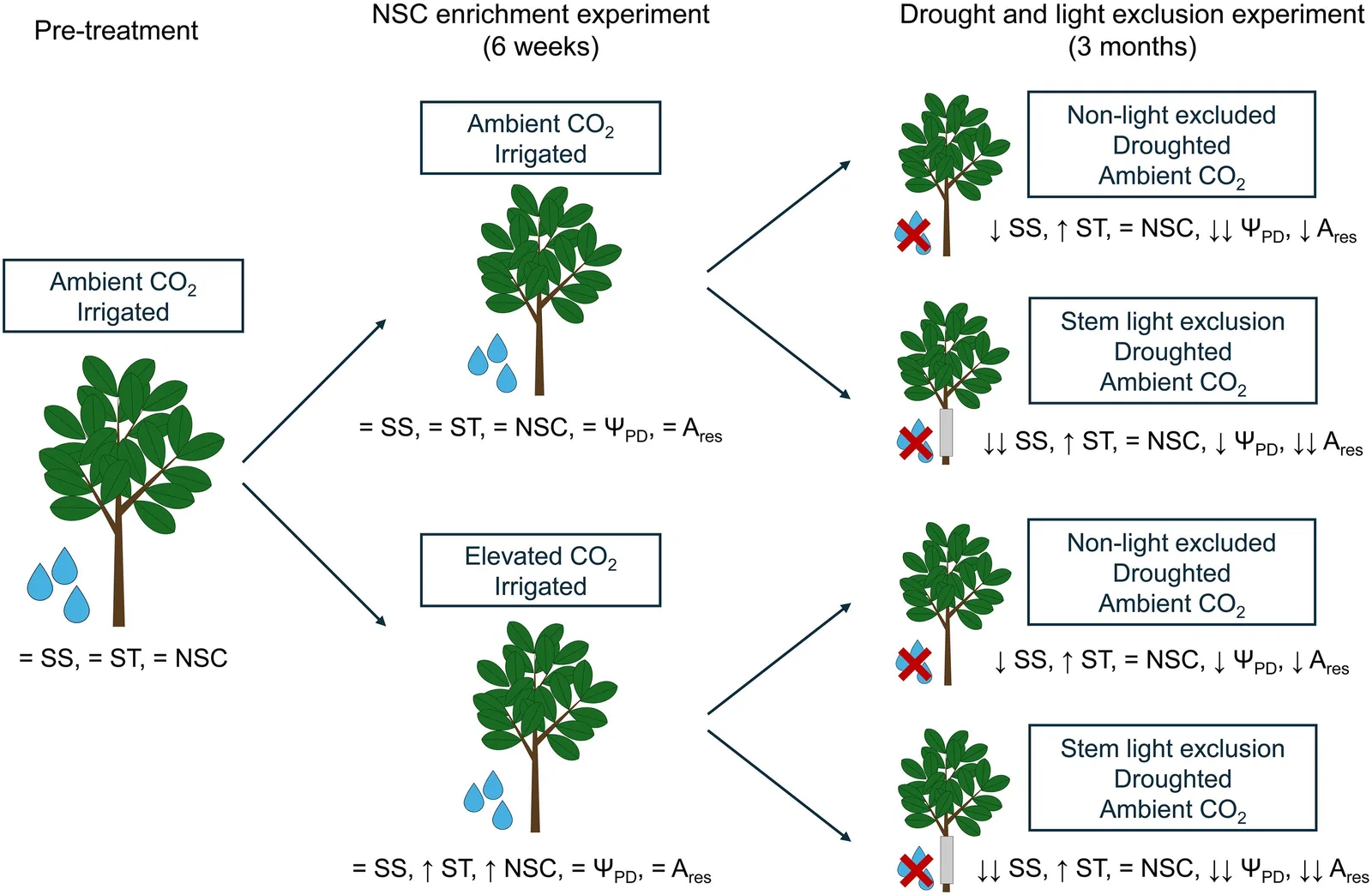
Experimental design and main results from Ávila-Lovera et al (2026). A_res_, stem reassimilation rate; SS, soluble sugar concentration; ST, starch concentration. Up-facing arrows (↑) indicate increases compared to pretreatment values, down-facing arrows (↓) indicate decreases, and an equal symbol (=) indicates no change. Double arrows indicate that the change was greater in that treatment than in the other ones.

As no other traits were affected by elevated CO_2_ levels, the NSC enhancement treatment allowed the authors to test whether trees with higher carbon reserves were able to better endure drought. They subjected all the plants (NSC enhanced and control) to a drought experiment for 3 months (until all plants died). Additionally, half of the plants in each group had their stems covered to deprive them of light and hence discern the roles of predrought NSC reserves and stem photosynthesis on drought-induced mortality (Fig. 1). Leaf photosynthetic rates and g_s_ decreased as drought progressed in all treatments, although g_s_ was higher in plants that had been exposed to higher CO_2_ concentrations. Conversely, specific leaf area (SLA = leaf area/leaf dry mass) was higher in trees subjected to elevated CO_2_ for an equivalent leaf area to control trees, suggesting a stronger investment in thicker, denser leaves to reduce water loss and thus better bear dry conditions in NSC-enhanced trees. Despite this observation, Ávila-Lovera and colleagues (2026) surprisingly found no significant differences between CO_2_ levels in the rate at which 100% plant death occurred, indicating that having greater NSC reserves predrought did not translate into longer survival under drought. Soluble sugars decreased and starch increased in leaves, stem wood, and, particularly, stem bark as drought progressed across treatments. Growth has been found to be more limited under drought than photosynthesis, leading to carbon accumulation in the form of starch despite decreasing photosynthetic rates (Huang et al 2021). At the time of death, saplings still had high NSC reserves, indicating that carbon starvation did not occur (Ávila-Lovera et al 2026). The significant drop in Ψ_PD_ as drought progressed may, however, point to hydraulic failure as the key mortality driver.

The authors found that *C. longifolium* stems and branches mostly perform stem recycling photosynthesis, rather than using external air as the source of CO_2_. Light exclusion reduced stem reassimilation rates, with no differences between CO_2_ exposure levels and reduced soluble sugar concentrations in leaves and stem bark compared to light-exposed trees. Yet, while stem photosynthesis was expected to decrease carbon losses and improve plant capacity to maintain xylem hydraulic integrity through osmoregulation, there was no effect of light exclusion on plant water status (ie, Ψ_PD_) or the rate of leaf or plant mortality. The authors thus propose that although stem photosynthesis may allow *C. longifolium* to maximize light interception and photosynthetic carbon assimilation when dry-season deciduous species shed their leaves, the resulting increased sugar availability does not play a significant role in plant survival under drought.

This study represents the first test of the carbon starvation hypothesis on trees with the capacity to perform stem photosynthesis and the role that this potential additional carbon source plays in plant survival under drought in tropical forest ecosystems. Further research incorporating this and other factors affecting NSC source and sink dynamics and comparing plants with different NSC reserve levels predrought is still needed to elucidate the impact carbon pools have in prolonging plant survival under drought and their feedback with hydraulic function.

**Recent related articles in *Plant Physiology*:**

- Alongi et al (2026) found that soluble sugar concentrations increased and starch concentrations decreased under severe drought in Douglas fir saplings but rapidly reached control values following rewatering, demonstrating that tree recovery after drought is mainly driven by stem hydraulic limitations.
- Waite et al (2026) found, using a model, that the mechanisms behind tree drought responses can be divided into a first phase that lasts until stomatal closure, which dominates the desiccation process and is closely associated with xylem vulnerability to embolism, while a second phase that extends until reaching lethal xylem hydraulic thresholds is influenced by water storage and residual conductance traits.

## Data Availability

No new data were generated or analyzed in support of this article.
